# The ill physician who self-discloses: What do patients think?

**DOI:** 10.1080/13814788.2022.2146091

**Published:** 2022-12-01

**Authors:** Barry Knishkowy, Noga Guggenheim

**Affiliations:** a Maccabi Healthcare Services, Mevasseret Tzion, Israel; b Aarburg, Switzerland

**Keywords:** General practice/family medicine, general patient involvement (empowerment self-management), communication

## Abstract

**Background:**

Physicians with a serious illness face difficult decisions about revealing this sensitive information to patients. Self-disclosure of illness is a largely unexplored topic, particularly from the patient’s perspective.

**Objectives:**

To learn about patients’ emotions and reactions to their family physician’s sharing with them about having a major illness.

**Methods:**

The study was carried out in a family practice office in a suburb of Jerusalem, beginning the day that a family physician returned to work after a prolonged illness. A questionnaire study was performed with nine closed and four open questions relating to patients’ reactions to learning about the illness. The questionnaire was distributed to 200 consecutive patients ages 18 years or older. Data extraction, compilation, and content analysis were performed to elicit and categorise major themes and issues that arose.

**Results:**

A total of 82% of the patients were pleased that the physician shared the information with them and none were displeased. Patients expressed a wide range of reactions to being told of the illness by the physician himself, among them: empathy, surprise, appreciation, pride, criticism, comfort/discomfort, and closeness. The value of sharing personal experience and the unique connection with the family physician were emphasised.

**Conclusion:**

Physician self-disclosure of major illnesses to patients can reveal the physician’s humanity, encourage empathy on the part of patients and strengthen the physician-patient relationship. This report adds to current knowledge about when to share this powerful information with patients and highlights the topic’s importance in the education of future doctors.

KEY MESSAGES

- Physician self-disclosure of major illness can elicit a wide range of emotions in patients, and often strengthens the physician-patient relationship.
- The vast majority of patients in this study were in favor of sharing such information with them.

## Introduction

Physician self-disclosure is often used intentionally, to establish rapport, foster trust and reciprocity, express empathy, provide hope and reassurance or enhance the ability to make credible recommendations. However, there may also be negative consequences, including taking away time from the patient’s visit, changing the focus of the visit, burdening the patient with the doctor’s problems, and even role reversal [1–9].

Revealing one’s health status is a unique type of self-disclosure. It is often unavoidable, colours the physician-patient relationship, and may have significant ramifications for the physician.

Physicians who experience a severe illness face the complex decision of whether to inform their patients about their health status. Sharing such personal, sensitive information with patients may have a powerful impact on the physician, the patient, and the nature of their relationship [10,11].

While physician self-disclosure on various issues has been addressed in the literature, very little is known about patients’ reactions to such revelations. Specifically, patients’ reactions to learning that their doctor is ill have never been studied systematically.

In September 2017, one of the authors (BK) underwent septal myectomy for advanced hypertrophic cardiomyopathy. He was absent from clinical work for six weeks. Upon return, colleagues, friends and family gave a range of divergent opinions on whether it was correct to openly and honestly report to patients about what had transpired. In the absence of any evidence-based recommendations, he decided to inform every patient who came for a visit during the period after his return and to ask for their reactions to being told. This paper is an analysis of their written responses.

This study aimed to learn about patients’ emotions and reactions to their family physician’s sharing with them about having a major illness.

## Methods

### Setting and population

The study was carried out in a family practice office in a suburb of Jerusalem between November 12 and December 3, 2017. The town’s population numbers about 18,000, is middle class and has a mixture of religious and secular Jewish residents. The participants received care at the only local branch of the Meuhedet Health Services HMO, where the author (BK) and one other family physician were the primary care staff.

There had been no advance notice to patients that the physician would be absent for an extended period. During the physician’s absence, secretaries told patients who came to the clinic that he was in the United States for a prolonged family visit.

### Study design

We carried out a questionnaire study of both closed and open questions in a sample of patients. The two authors drafted the questionnaire (Appendix) independently and then together. It was piloted with eight men and women of various ages and then finalised. It included demographic data, followed by nine closed and three open questions about reactions to and opinions regarding learning of the physician’s illness and his sharing this information with patients. We included the open questions to explore an additional, more personal dimension of the observed phenomenon. In these questions, we asked participants to freely describe their thoughts and feelings after being informed that the physician had been ill, and how they felt about the fact that the doctor himself had informed them about his illness. A fourth open question added mid-survey, asked patients what they would think or feel if the doctor’s illness had not improved. This report focuses principally on the study’s open questions.

### Data collection

The study began on the day that the physician returned to work. The physician told each patient who mentioned the absence: ‘I was out of the country because I had to have heart surgery for an unusual, genetic condition that could not be repaired in Israel. The operation was successful, and now I am feeling well.’ If the patient did not raise the issue, this sentence was prefaced with: ‘Did you know that I was not at work for a very long time?’. The topic was presented in an optimistic tone, and patient questions were answered straightforwardly. The focus of the discussion then switched quickly to the patient’s reason for visitation.

At the end of the consultation, the physician asked the patient if s/he would be willing to fill out an anonymous questionnaire regarding the doctor’s severe illness. The questionnaire was then given to the patient by the physician himself, to be filled out in the waiting room and returned to the secretary. IRB approval was not requested, as the data collected were anonymous and did not include information directly relating to the health or medical treatment of the patients.

The questionnaire was distributed to 200 consecutive patients 18 years of age or older who attended the clinic during the study period. The last 98 patients (103–200) received the questionnaire with the fourth open question.

### Data analysis – open questions

Anonymous data extraction and compilation of the open questions were performed by one of the authors (NG). Content analysis of the first 20 questionnaires was then performed and discussed by both authors to elicit primary themes and formulate categories. The next phase entailed a dynamic and ongoing process of analysing the entire body of questionnaires, identifying additional themes. This involved constructing tables separately for each question that included: the questionnaire number with demographic data; 'significance units’ – word for word patient responses (e.g. ‘I was both sad and happy’); 'coding’ (e.g. using the method of marker words and sentences); author’s comments (e.g. search specifically for expressions of ambivalence in a specific age group); 'categories’ (e.g. emotions); 'themes’ (e.g. mixed feelings or ambivalence). All respondents’ data was organised and analysed according to gender, age and marital status. An ongoing dialogue between the researchers facilitated mutual exposure to their thoughts and feelings [12,13].

## Results

A total of 200 patients visited the physician during the study period, 165 (82.5%) returned the questionnaires. Patient characteristics are presented in Table 1.

**Table 1.** Patient characteristics (*n* = 165).

Mean age, years [SD]	45.9 [16.5]
Gender *n* [%}	
Male	58 [35.2]
Female	107 [64.8]
Marital Status n [%]	
Married	120 [72.7]
Divorced	16 [9.7]
Living Separately	2 [1.2]
Single	25 [15.2]
Other	2 [1.2]
Religiosity (Jewish) *n* [%]	
Ultra-Orthodox	20 {12.1]
Orthodox	43 [26.1]
Traditional	41 [24.8]
Secular	61 [37.0]

### Brief summary of the closed questions responses

20% of the patients felt that after being absent due to a major illness, a physician should tell all patients the reason, 55% that he should only tell those who inquire, 7% that he should give a different reason to those who ask, and 18% had no opinion or gave a different response. In total, 82% were very pleased that the physician shared the information with them, 12% were somewhat pleased, 6% responded that 'it doesn’t matter’, and none were displeased.

### Content analysis of the open questions’ responses

The themes that were formulated stemmed from the content analysis. They will be presented with a description and a limited selection of representative citations. No significant differences in replies according to the demographic variables were identified.

### Thoughts and feelings about learning of the physician’s illness

In this section, we received many emotional responses from the patients. Identification with the physician was another major theme. A small number of responses were matter-of-fact and non-emotional.

### Themes

#### Emotional reactions

There was a wide range of emotional responses. Sadness, concern and pain were the most common. Following these in order of frequency were expressions of surprise and even shock at learning that the doctor had been ill, often combined with concern or worry about his well-being and health. There were also moving responses that expressed happiness and relief that everything had gone smoothly and that the physician had returned to work as usual, several with compliments to this particular doctor. Many statements combined several emotions, such as sadness together with happiness about the recovery.

*On the one hand, I was very sorry to hear about this, and on the other, was happy that he is feeling well.* (Q132)

#### Physician as a human being

A widespread reason for feeling surprised was the very prevalent perception that doctors, by the nature of their profession, do not or should not become ill themselves.

*I was a bit surprised because we’re talking about a doctor, and sometimes it seems that doctors don’t get sick because they know how to take care of themselves.* (Q131)

The realisation that the physician is a human being like everyone else and that there is a common human denominator was philosophically expressed by some.

*I'm not the only one with pain and feelings or poor health. We all have inherited genetic problems. We should know that the doctor is no different from us. He suffers pain and fears just like I do. Sometime he needs a doctor to take care of him.* (Q36)

### Thoughts and feelings about the physician’s self-disclosure

In this section, we asked the respondents to examine their thoughts and feelings that arose, not from learning of the doctor’s illness but from a unique and unconventional experience: that the person giving the notification of the health condition was the physician himself who had become ill. This subject elicited a significant number and wide range of responses. We note that most patients favoured their physician’s sharing information with them about having a serious illness – matching the findings in the quantitative analysis.

### Themes

- *Feelings towards the physician and reactions to the revelation*

#### Empathy

The experience of shared solidarity and human fragility was expressed in many responses.

*I identify with the difficulties that he went through, and just as I feel that he worries about me, so I worry and think about him.* (Q44)

#### Surprise

Surprise was a widespread reaction among the respondents, who were divided between those who simply gave a one-word response – ‘surprise;’ and those who expanded on their surprise that the doctor had opened up to them about his disease.

*I felt surprised but it gave me a good feeling and a sense of closeness, in that if he tells me about this and shares something so personal with me, this reveals humanity, openness, frankness.* (Q26)

#### Appreciation

In general, we have the impression that the status of openness and exposure was much appreciated. Most of the expressions of appreciation were brief (‘brave,’ ‘congratulations to him’), but some wrote more explicitly about the directness, courage and sincerity and how this affected them.

*I deeply respect the doctor’s decision to tell his patients about his illness… I appreciate him even more because of this. Such a step requires great courage and openness.* (Q157)

#### Pride

Another common and notable reaction that was experienced as a result of this unexpected and unique encounter with the physician was pride, feeling worthy of mutual trust and important, in that the physician had chosen to share this very personal information with him.

*I'm proud that he was willing to open his private self and share with me.* (Q36)

*I was happy. This allowed me to feel that the doctor trusts me enough as a human being to tell me what he had gone through, just as I trust him.* (Q192)

#### Criticism

Reasons for criticism included a feeling that the physician should not share such information with patients.

*… he doesn’t have to publicise this to every patient. This is a personal matter.* (Q136)

*I was surprised but mainly felt that this wasn’t so much my business.* (Q180)

As well as quite the opposite, he should have informed patients of the illness in advance.

*I would have wanted to hear about this before he travelled for the surgery, so that at least I could have wished him a full recovery… (Q159)*

#### Compassion and concern

Beyond empathy, several patients felt compassion towards their now-vulnerable doctor. Sadness and concern for the doctor’s well-being were also often noted. However, very few respondents expressed pity or worry about his ability to function. The experience of actually seeing the physician facing them and appearing perfectly healthy undoubtedly affected their responses, minimising such reactions.

#### Laconic, unemotional reactions

We were impressed that one-word responses, clearly expressing a lack of emotion or thought, such as ‘normal’ or ‘regular’ were very uncommon, as were statements showing that the patient was not particularly moved. For example, 'nice’ was expressed by two very young participants.

b.*Feelings during the encounter*

#### Comfort/discomfort

Many participants spoke of feeling comfortable with the fact that the doctor had shared personal medical information with them.

*I felt very comfortable, nice on his part and warm.* (Q15)

However, a few explicitly expressed discomfort, embarrassment or mixed feelings. We must learn from this that there were also reservations about the directness and openness of the doctor.

*Initially, I thought it was a bit weird that he was sharing this with me but ultimately I appreciated his sincerity and sharing. His sharing caused me to feel more at ease.* (Q3)

*I felt embarrassed, unsure that this was any of my business. I appreciate privacy.* (Q103)

For some patients, discomfort or ambivalence was due to their respect for the physician’s right to privacy.

*I hope that he understood, that I didn’t ask about his absence out of respect for his privacy, and not out of indifference.* (Q33)

c.*Impact on the physician-patient relationship*

#### Value of personal experience

The value of sharing personal experiences was expressed multiple times as a feature that adds value to the physician. Patients perceived that a physicians’ age and experience, including the personal experience of illness, add to their ability to understand, advise, empathise and inspire trust.

*This strengthens the relationship and creates a feeling of trust between physician and patient…Only in this way does the patient feel more secure and involved and less uncertain.* (Q8)

*The physician’s ability to honestly share with his patients not only his professional knowledge and experience, but also his personal experience… It strengthens and inspires confidence! …. A doctor who knows first-hand what a disease is and the feelings that accompany it can be more empathetic and sensitive to patients.* (Q125)

#### Engenders closeness

Needless to say, several respondents expressed the feeling that sharing personal information with them heightened their feeling of closeness with the physician.

*This can draw the patient closer to the doctor and improve the communication between them.* (Q130)

#### Unique connection with family physician

Finally, we found several noteworthy responses that distinguished Family Medicine from other specialties. The longstanding, multifaceted relationship with patients that develops with family doctors seemed to nurture a deep, bidirectional connection that may not be present with other specialities.

*It’s important for me to know, not because I'm curious but rather because I feel an important connection specifically with my family doctor, beyond what I feel for other kinds of doctors.* (Q152)

*The relationship between a family doctor and a patient are generally over many years and so it’s important to me… that the doctor connects to me beyond tests and medications…that the doctor shares his feelings and his illness.* (*Q44)*

### Hypothetical reactions if the physician had not recovered

We included a hypothetical question to understand whether the patients could share their thoughts and emotions about ‘what would happen if?.’ Most participants who chose to answer this question (76% of the subsample) selected one-word emotional responses, usually a non-quantifiable noun (e.g. sadness, worry, compassion, sorrow), and avoided verbal inflexion in the first person. This lack of content stood out, particularly in the questionnaires of participants who answered the other questions in considerable detail. It probably reflects significant patient discomfort in acknowledging or dealing with this scenario.

## Discussion

### Main findings

The significant finding of this study was that these encounters elicited and demonstrated a wide range of moving responses related to sharing an emotionally laden human experience with the family physician. Patients expressed sadness, identification and an enhanced sense of closeness. Noteworthy were the feelings of pride that the physician had chosen to confide in them and having the opportunity to show compassion towards the person who is usually the source of compassion.

Nearly all patients were very pleased that they had been informed about the illness and were particularly touched by experiencing the face-to-face revelation by the physician himself. On the other hand, while no patients replied that they were displeased by the disclosure, very few did express discomfort about being in this situation. While most patients in this population agreed with telling only those who asked, a significant minority felt that all patients should be informed. Intuition would have it, and the physician himself ultimately felt it appropriate to share this intimate information only with veteran patients or those who inquired.

Personal experience as a patient deepens the practitioner’s understanding of illness and its ramifications, often fostering empathy and the ability to be a mentor and guide. Practitioners may exploit it as an educational and motivational tool [5,8,9,14]. From the patient’s perspective, such experience may enhance the physician’s value, inspiring confidence that cannot be obtained in formal training and is less likely to be felt with younger, less-weathered doctors.

The value of family medicine as a unique speciality was explicitly and implicitly expressed throughout this collection of moving responses. The characteristically long-term, multifaceted relationships which develop undoubtedly have rich consequences. These responses exposed the deep and perhaps unrecognised emotional bonds that may form between patients and their family physicians.

### Strengths and limitations

This paper is unique in that it gives patients’ views on physician self-disclosure about being ill, utilising a systematic and uniform methodology. It provides information not accessible from previous surveys or interviews with the physicians themselves, or from researchers’ analyses of visits to doctors.

Numerous factors most certainly influenced patients’ reactions: the actual diagnosis, the physician’s specialty, the closeness of the relationship, the length of time under the physician’s care, the setting in a small community, and the physician’s own personality and style of communication. The results are therefore not generalisable to other populations.

The study’s open questions provided particular strengths regarding the depth of information it provided about this specific and unique phenomenon. However, there were also limitations. Conducting interviews – a more optimal way of obtaining data – was impossible for lack of time. Additional identifying characteristics, such as time cared for by the physician, might have given greater insight into determinants of the patients’ responses.

The primary source of bias in this study was the fact that the study was carried out by the same physician who cared for the patients being surveyed. Many patients with a longstanding relationship might not have wanted to upset a close relationship. This may have resulted in overly positive results. Possible patient concerns about lack of anonymity, especially with open questions, may have added to this problem. Nevertheless, the patients were promised and given absolute confidentiality and no identification in the data analysis. Another potentially significant source of bias was the 18% nonresponse rate. Many of these patients, specifically may have been critical of the doctor’s sharing information.

### Comparison with existing literature

A study in which 50 physicians with HIV and other major illnesses were interviewed illuminates the feelings, struggles and dilemmas of these men and women, transformed into the role of physician-patient. Many of these physicians elected to self-disclose because of the discomfort in concealing or the conviction that their experience with illness and treatment could benefit their patients. Others decided against being open, feeling that disclosure was irrelevant or could threaten the relationship’s stability, lead to stigmatisation or harm them professionally [10,11]. Essays by physician–patients caring for patients with the same diagnosis have poignantly described both the tangible benefits and the potential dangers of sharing their stories with patients [14,15].

The current study is the first (to the best of our knowledge) that specifically and systematically examines the patients’ reactions to learning that their physician has a major illness and to self-disclosure about the illness by the physician. While to some extent confirming the impressions and beliefs of physicians and researchers in previous reports, this patient-oriented study reveals a rich world of patient experience and emotion that has not been previously described.

Discovering that the healer himself is a vulnerable, even mortal human being was an eye-opening revelation for many respondents. This possibility had not occurred to them and did not fit into the accepted paradigms of an omniscient professional who prevents and cures illness. Cognitive coping with questions about possible death is naturally threatening, especially when it concerns those in whom we entrust our health. While much has been written about the therapist’s vulnerability in the mental health professions [16,17], these patients’ remarks illuminate just how poignant these issues are regarding the medical profession as well.

The potentially negative consequences to the patient of physician self-disclosure, posited by several authors [10,11,18,19], were reflected in the comments of only a tiny minority in this sample. To some extent, this may be explained by the brief and reassuring manner in which the news was shared but it may also demonstrate that such concerns have been overstated.

Perceived or actual risk of self-disclosure to the impaired physician’s career has been the subject of previous works, most notably regarding mental illness and also conditions such as substance abuse, HIV and advanced malignancy [10,11,20–23]. To what degree the fear of disclosure is based on reality from the patient’s vantage point is not well known. In the current study, patients did not have to deal with the mixed feelings and dilemmas that would have arisen if the doctor had been perceived as posing a threat, unable to fully function, or having a more questionable future. It remains unanswered whether, under a different constellation of circumstances, their emotional responses would have been qualitatively different or whether they would have considered leaving the doctor.

### Implications for clinical practice and research

The study adds to current knowledge about when to share this powerful information with patients. It warrants further investigation and discussion, especially in the education of future doctors.

## Conclusion

Physician self-disclosure of major illness to patients can potentially reveal the physician’s humanity, encourage empathy on the part of patients, and strengthen the physician-patient relationship.

## Appendix

Patient Questionnaire: English Translation of the Original Hebrew Text

Your family physician was recently ill with an illness that required a prolonged absence from the office. We are interested in understanding your thoughts and feelings about this, and we thank you for your answers. This anonymous questionnaire will take about five minutes of your time.

Sincerely,

The Office Staff

Questionnaire Number ____

Date __/__/__ (day/month/year)

Gender: Male/Female

Age (in years): ____

Religiosity: Ultra-Orthodox/Orthodox/Traditional/Secular/Other ____

Marital Status: Married/Divorced/Living Separately/Single/Other ____

1. From whom did you hear that the doctor was ill and had an operation? (circle all of the correct answers)
  - from the doctor himself
  - from another doctor in the office
  - from a nurse in the office
  - from the office manager or a secretary
  - from another patient
  - other _______
2. When did you hear about this?
  - today
  - before today
3. If you didn’t talk to the doctor about his illness during the visit, what was the reason? (circle all the appropriate answers)
  - I didn’t know that he hadn’t been at work.
  - I thought this was none of my business.
  - I was embarrassed.
  - I didn’t think it was important.
  - other __________
4. How did you feel after hearing about the doctor’s illness and the operation he underwent? (circle all the appropriate answers)
  - surprised
  - shocked
  - sad
  - angry
  - uncomfortable
  - I didn’t feel anything special.
  - other __________
5. Before you were told the reason for the doctor’s absence, what did you think was the reason?
  - illness
  - a family member’s illness
  - vacation
  - I didn’t think about it.
  - other __________
6. In your opinion, when a doctor has a significant illness, is it right to share this information with patients?
  - yes
  - no
  - I have no opinion about this.
7. In your opinion, when a doctor needs to be away from work for a prolonged time because of illness, before the absence s/he should: (circle one answer)
  - tell patients that s/he will be away, with the reason for the absence
  - tell patients that s/he will be away, but not tell the reason
  - not say anything
  - I have no opinion about this.
8. In your opinion, when a doctor needs to be away from work for a prolonged time because of illness, after s/he returns to work s/he should: (circle one answer)
  - tell the true reason for the absence, only to patients who ask
  - tell a different reason for the absence, and only to patients who ask
  - tell the true reason for the absence to every patient
  - tell a different reason for the absence to every patient
  - I have no opinion.
9. What is true for you? (circle one answer)
  - I am very pleased that the physician told me what he went through.
  - I am somewhat pleased that the physician told me what he went through.
  - It doesn’t matter that the physician told me what he went through.
  - I am somewhat displeased that the physician told me what he went through.
  - I am very displeased that the physician told me what he went through.

For the following questions, we would appreciate a detailed response, to the extent possible.

10.Describe in your own words the thoughts and feelings that you had after you learned that the doctor was ill.
11.Describe the thoughts and feelings you had regarding the fact that the doctor himself told you about his illness.
12.Tell with whom you shared the thoughts and feelings that arose, and what the responses were.
13.If the doctor’s illness had not improved, how would this have influenced your thoughts and feelings towards him?
